# Efficacy of surgery for the treatment of astrocytoma: A protocol of systematic review and meta-analysis

**DOI:** 10.1097/MD.0000000000020485

**Published:** 2020-06-05

**Authors:** Guo-wei Wang, Bao-ming Li

**Affiliations:** a Department of Neurosurgery, Weibei Central Hospital, Weinan; b Department of Neurosurgery, No.215 Hospital of Shaanxi Nuclear Industry, Xianyang, Shaanxi, China.

**Keywords:** astrocytoma, complications, efficacy, surgery

## Abstract

**Background:**

This study will assess the efficacy of surgery for the treatment of patients with astrocytoma.

**Methods:**

We will undertake searches for randomized controlled trials from the following databases: MEDLINE, EMBASE, Cochrane Library, CINAHL, PsycINFO, and China National Knowledge Infrastructure. We will search all these databases from their inception to the March 1, 2020. No language limitation and publication status will be imposed in this study. Two authors will independently carry out study selection, data extraction, and study quality assessment. We will invite another author to solve any divergences between 2 authors. We will use RevMan 5.3 software to conduct statistical analysis.

**Results:**

This study will present synthesis of most recent evidence of surgery for the treatment of patients with astrocytoma.

**Conclusion:**

The findings of this study will provide helpful reference for the efficacy and complications of surgery for the treatment of patients with astrocytoma to the clinicians and future researchers.

**Study registration:**

INPLASY202040194.

## 1 Introduction

Astrocytoma is one of the most common cancers in brain and spinal cord,^[1–5]^ which accounts for about 30% of all primitive intramedullary tumors with cervicothoracic predominance.^[6]^ It occurs in astrocytes that support nerve cells, and its signs and symptoms’ manifestation is based on its location.^[7–10]^ During the past decades, surgery is regarded as the best choice to treat astrocytoma.^[11–21]^ However, there is no systematic review or meta-analysis providing evidence to determine whether surgery is an ideal therapy. Therefore, this study will assess the efficacy and complications of surgery for patients with astrocytoma, expecting to provide high-quality evidence to support clinical practice.

## 2 Methods

### 2.1 Study registry

This study has been registered on INPLASY202040194. We will utilize the Preferred Reporting Items for Systematic reviews and Meta-analyses Protocols statement to guide this study.^[22]^

### 2.2 Eligibility criteria

#### 2.2.1 Type of studies

This study will only consider randomized controlled trials that assess the efficacy and complications of surgery for the treatment of patients with astrocytoma.

#### 2.2.2 Type of participants

All patients who were diagnosed as astrocytoma will be considered for inclusion in this study. We will not apply any restrictions of race, age, sex, education background, and economic status.

#### 2.2.3 Type of interventions

All subjects in the experiment group underwent surgery for the treatment of astrocytoma.

All patients in the control group who received any types of therapies will be included in this study.

#### 2.2.4 Type of outcomes

The primary outcomes include overall survival and pathological complete response. The secondary outcomes consist of recurrence-free survival; disease-free survival; quality of life, as assessed any Health-Related Quality of Life scales; and complications.

### 2.3 Search strategy

MEDLINE, EMBASE, Cochrane Library, CINAHL, PsycINFO, and China National Knowledge Infrastructure will be searched from their inception to the March 1, 2020. We will not impose any limitations to language and publication status. The search strategy will be built with the assistance of a professional librarian. The provisional MEDLINE search strategy is presented in Table 1. We will modify similar search strategies to the other electronic databases. Additionally, we will search Google scholar, conference abstracts, and reference lists of included studies to avoid missing any potential eligible trials.

**Table 1:**
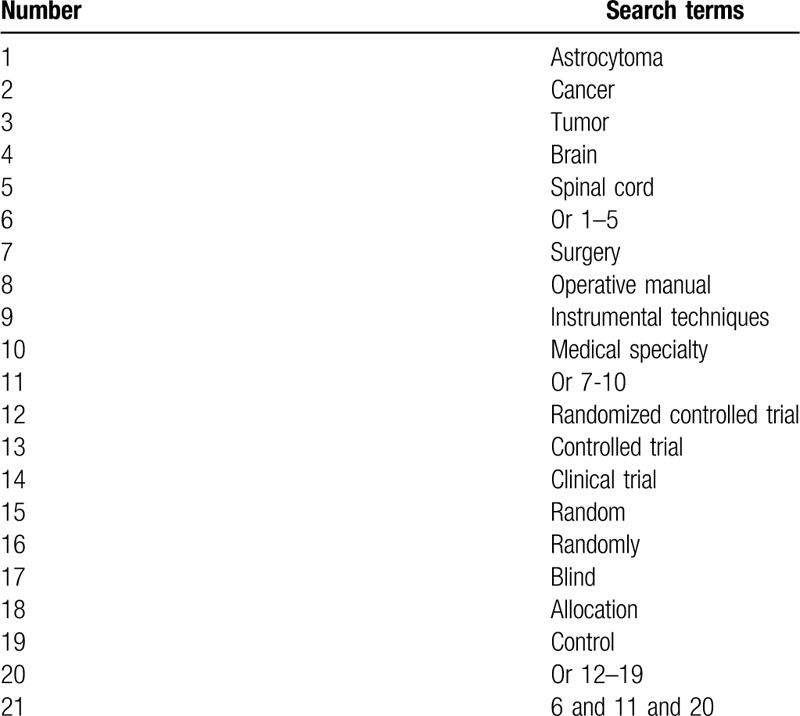
Search strategy sample of MEDLINE.

### 2.4 Data selection and extraction

#### 2.4.1 Study selection

Before selecting literature, we will perform training and a calibration exercise within review team. All searched studies will be imported to Endnote 7.0, and all duplicated or irrelevant studies will be removed. Then, we will identify titles/abstracts of all literature by 2 independent authors. If necessary, full texts of potential studies will be obtained to further check their eligibility. Disagreements between 2 authors will be solved by another author through consultation. We will present the procedure of study selection in the flow diagram.

#### 2.4.2 Data extraction

All essential data will be extracted using previously created data collection sheet by 2 independent authors. Discrepancies in data collection between 2 authors will be settled down through discussion with the help of another author. We will extract the following information: study characteristics (first author, year of publication, country, study setting, and so on), participants (race, age, sex, eligibility criteria, and so on), study methods (sample size, randomization, blind, and so on), details of interventions and controls (types of treatment, delivery method, duration, frequency, dosage, and so on), outcomes (all primary and secondary outcome measurements, complications, and so on), and funding details.

#### 2.4.3 Risk of bias assessment

Two independent authors will assess all risk of bias for each study using Cochrane Risk of Bias Tool, regardless their methodological quality. It rates selection, performance, detection, attrition, reporting, and other bias for each included study. Then, each item will be further graded as low, unclear, or high risk of bias. All disagreements will be solved through discussion by another author.

### 2.5 Data synthesis and analysis

Synthesis of the data will be undertaken using RevMan 5.3 software. All quantitative data will be expressed using mean difference or standardized mean difference and 95% confidence intervals (CIs). All dichotomous data will be calculated using risk ratio and 95% CIs. We will use *I*^*2*^ index to assess the proportion of heterogeneity among studies. The values of *I*^2^ ≤50% show acceptable homogeneity, and we will exert a fixed-effects model for data pooling. However, the values of *I*^2^ >50% mean significant heterogeneity, and we will employ a random-effects model for data synthesizing. If it is possible, we will perform meta-analysis to analyze the pooled outcome data when acceptable homogeneity has been identified. Otherwise, we will conduct subgroup analysis to investigate potential causes for substantial heterogeneity among eligible studies.

### 2.6 Subgroup analysis

We will carry out subgroup analysis according to the study characteristics, study methods, interventions, comparators, and outcomes.

### 2.7 Sensitivity analysis

We will operate sensitivity analysis to identify the robustness of merged outcome results by removing high risk of bias studies.

### 2.8 Reporting bias

If >10 studies are included, we will perform Funnel plot^[23]^ and Egger regression test^[24]^ to check whether there exists any reporting bias.

### 2.9 Ethics and dissemination

We will not obtain ethic documents because this study will be conducted based on the data of published literature. We expect to publish this study on a peer-reviewed journal.

## 3 Discussion

Astrocytoma is a very common cancer in the central nerve system.^[1–5]^ During the past few decades, surgery is utilized to treat patients with astrocytoma.^[11–21]^ However, no study has published focusing on the systematic and comprehensive summary of the existing clinical evidence, which may restrict its application. In this study, we will perform a systematic review to summarize high-quality studies and to provide evidence on the evidence-based medical support for clinical practice.

## Author contributions

**Conceptualization:** Guo-wei Wang, Bao-ming Li.

**Data curation:** Guo-wei Wang, Bao-ming Li.

**Formal analysis:** Guo-wei Wang.

**Investigation:** Bao-ming Li.

**Methodology:** Guo-wei Wang.

**Project administration:** Bao-ming Li.

**Resources:** Guo-wei Wang.

**Software:** Guo-wei Wang.

**Supervision:** Bao-ming Li.

**Validation:** Guo-wei Wang, Bao-ming Li.

**Visualization:** Guo-wei Wang, Bao-ming Li.

**Writing – original draft:** Guo-wei Wang, Bao-ming Li.

**Writing – review & editing:** Guo-wei Wang, Bao-ming Li.
